# Supplementary material for: Cortical PV and VIP interneurons similarly influence SST neuron output despite distinct unitary properties
Source: Commun Biol. 2026 Jun 3;9:756. doi: 10.1038/s42003-026-10418-2 (PMC13234287; doi:10.1038/s42003-026-10418-2)
Supplement: Supplementary file 2 — Supplementary Information [file 42003_2026_10418_MOESM2_ESM.pdf]

Supplementary Figures

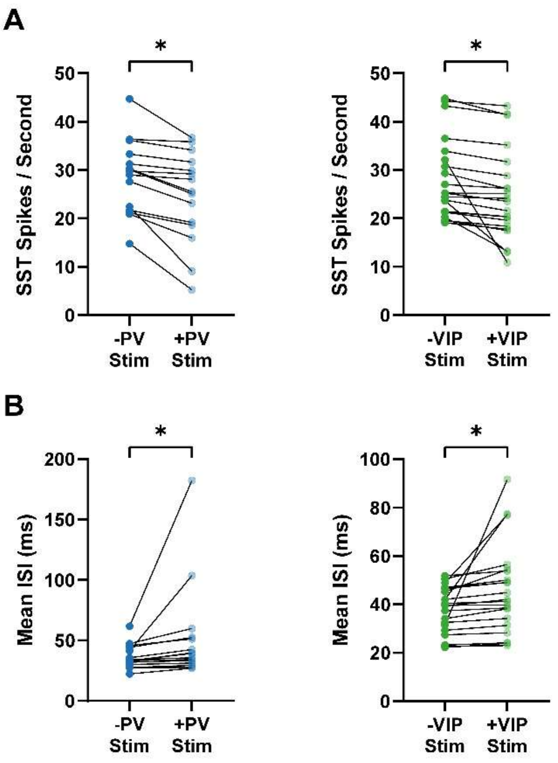

Figure S1: Presynaptic firing of PV and VIP cells leads to significant spike reduction and inter-spike-interval-increase in postsynaptic SST cells.

(A) Quantitative analysis of mean number of SST spikes with and without presynaptic stimulation of PV (blue, left) or VIP cells (green, right). (B) Quantitative analysis of mean ISI with and without presynaptic stimulation of PV (blue, left) or VIP cells (green, right). In both connections, presynaptic stimulation led to a significant decrease of SST action potential firing and a significant increase of inter-spike-interval length. Asterisks indicate significant differences between groups ($p<0.05$).

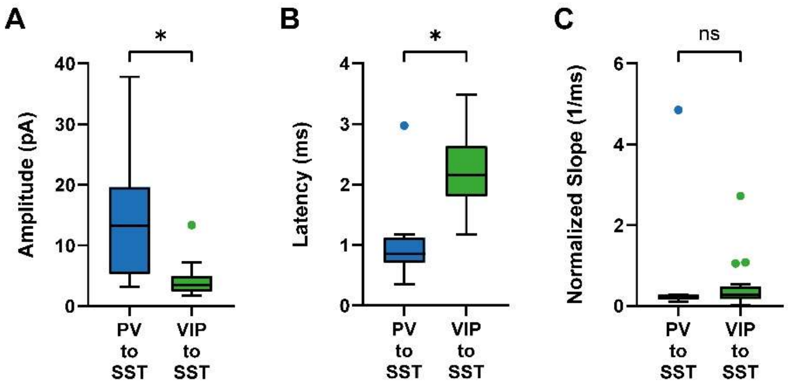

Figure S2: Unitary synaptic properties of PV to SST and VIP to SST cell connections reveal cell type-specific differences.

Quantitative analysis of the first IPSC recorded in PV to SST (blue) and VIP to SST (green) cell pairs. The following parameters have been analyzed: (A) amplitude, (B) latency, and (C) normalized slope. (A) PV to SST: n = 14, VIP to SST: n = 19, (B) PV to SST: n = 15, VIP to SST: n = 21, (C) PV to SST: n = 15, VIP to SST: n = 19. Asterisks indicate significant differences between groups ($p<0.05$).

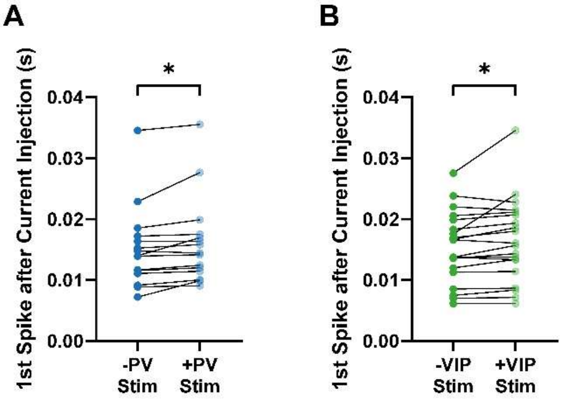

Figure S3: PV and VIP cells significantly delay firing onset of postsynaptic SST cells

Quantitative analysis of SST spike timing at the firing position named “early” after PV (A) and VIP (B) stimulation. Shown on the left of each graph are SST spike timings of the first spike without (left) and with presynaptic stimulation (right). In both motifs, presynaptic stimulation let to a significant delay of the first spike. Asterisks indicate significant differences between groups ($p<0.05$).

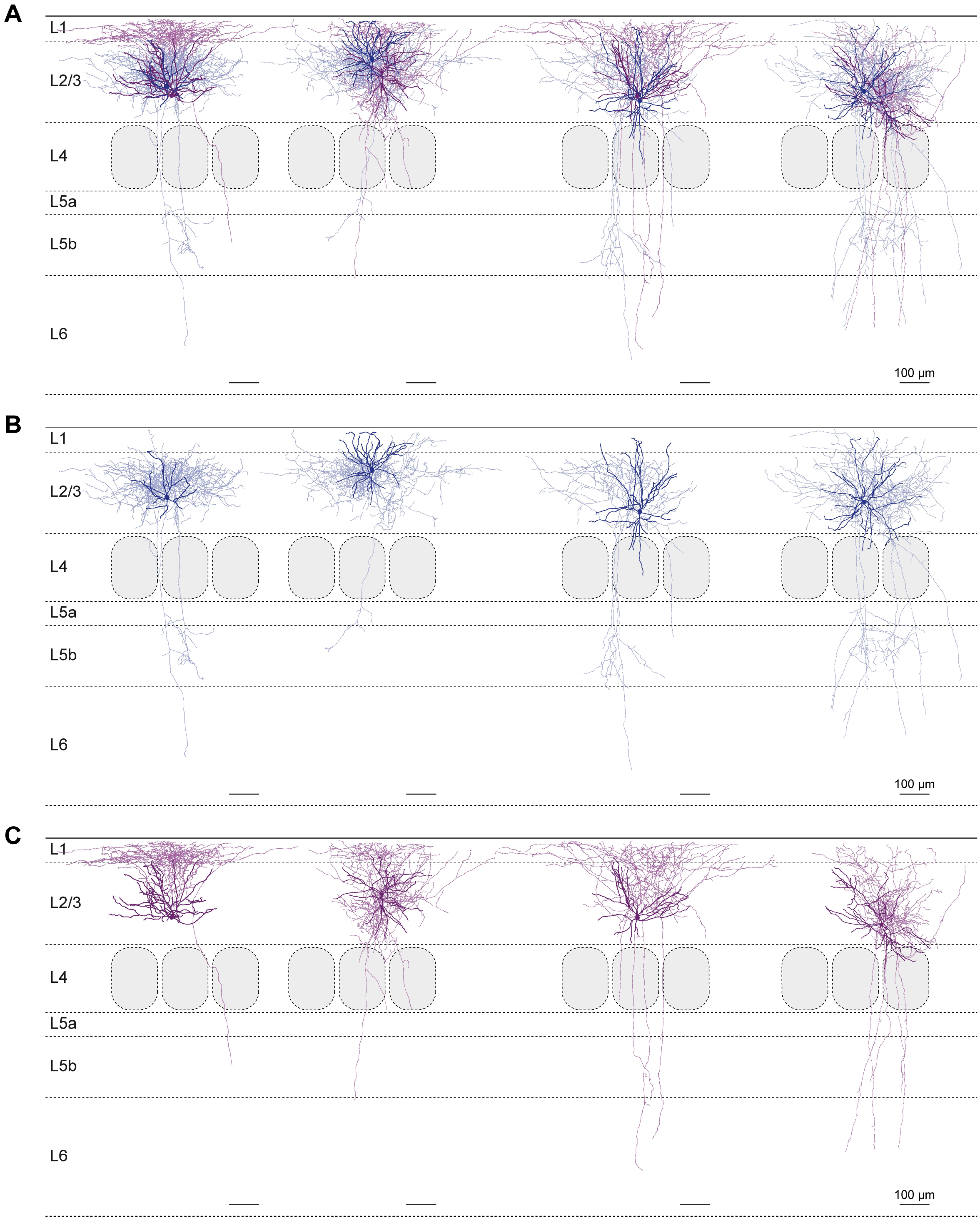

Figure S4: Gallery of morphologically reconstructed PV to SST cell pairs.

(A) PV cell soma and dendrites are shown in dark blue axon in light blue. SST cell soma and dendrites are shown in dark purple and axon in light purple. To better display the details of individual cells, PV and SST cells are shown separately in B and C. PV cells can be classified as BC and only show sparse or no axonal component in L1 (B). All SST cells display a strong axonal ramification in L1 and can therefore be classified as MC (C).

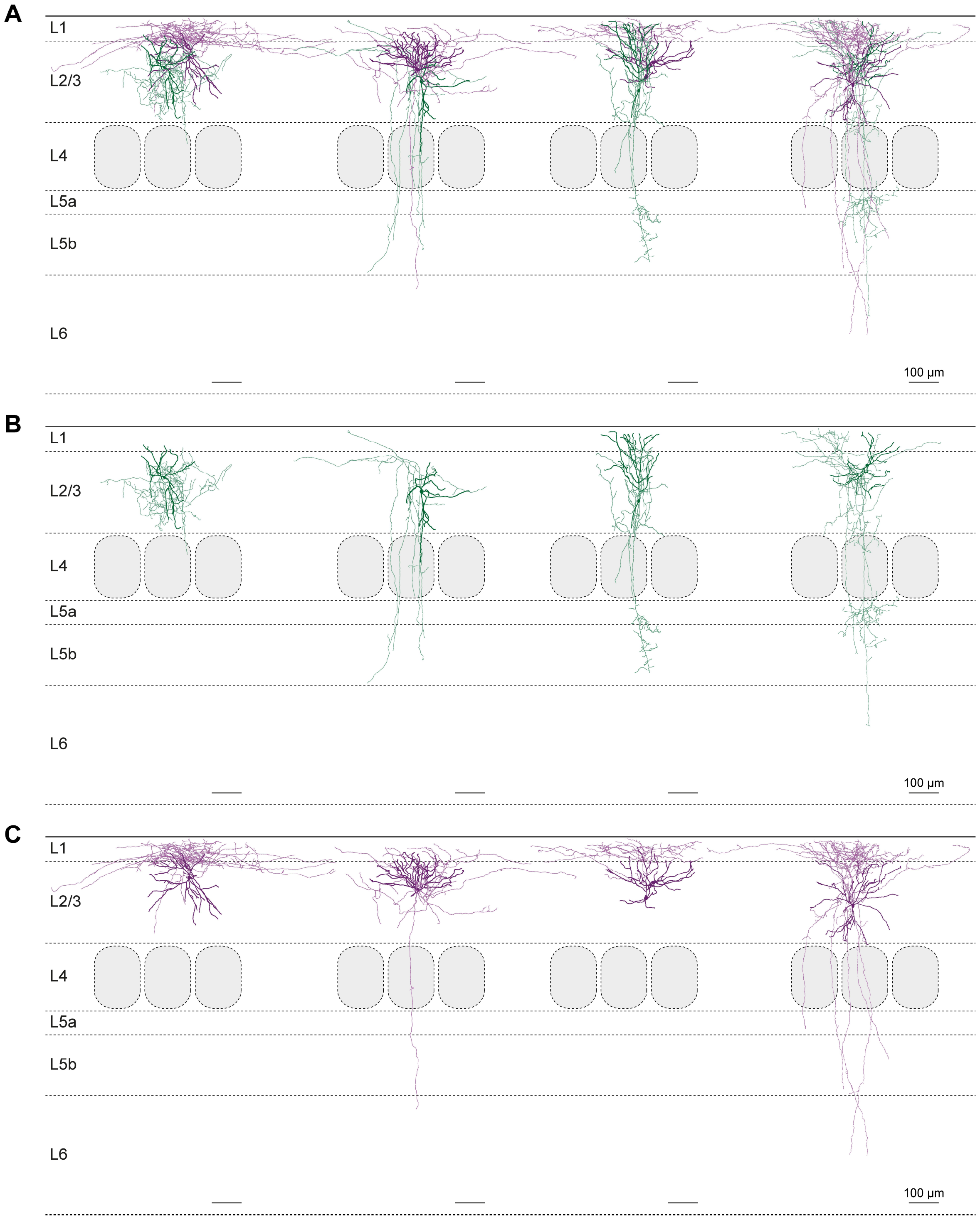

Figure S5: Gallery of morphologically reconstructed VIP to SST cell pairs.

(A) VIP cell soma and dendrites are shown in dark green and the axon in light green. SST cell soma and dendrites are shown in dark purple and axon in light purple. To better display the details of individual cells, VIP and SST cells are separately shown in B and C. The two VIP cells on the left can be characterized as multipolar cells. The most left VIP cell resembles a small basket cell. The two VIP cells at the right can be characterized as bipolar cells with a typical columnar ramification of the axon (B). All SST display a large axonal ramification in L1 and can therefore be classified as MC (C).

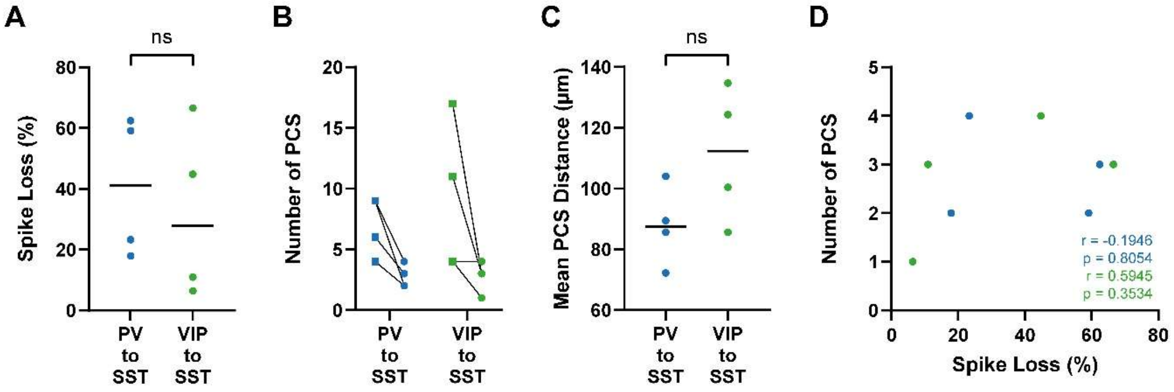

Figure S6: Supplemental information on PCS analysis.

Four pairs of each connection motif were morphologically reconstructed. (A) Reconstructed pairs displayed various effect strengths. Effect strength of both groups was not significantly different. (B) PCS number was identified during reconstruction (squares) and validated using enhanced confocal microscopy (circles). In 7 out of 8 reconstructed cells pairs, validation resulted in reduced numbers of PCS, thereby reducing the inherent tendency of overestimating PCS numbers using standard confocal microscopy. (C) Quantitative analysis of mean PCS distance from soma. Although PV and VIP cells are thought to target different subcellular compartments of their postsynaptic target cells, we did not observe a significant difference between both groups. However, the median of mean PCS distance of VIP to SST connections was ≈1.5 times larger than of PV to SST cell pairs. (D) Pearson correlation of long pulse spike loss and number of PCS of reconstructed PV to SST (blue) and VIP to SST cell pairs (green). Number of PCS was not correlated with spike loss in neither connectivity motif. The correlation coefficient is given as r. No significant correlations with $p < 0.05$ were found.

Table S1: Parameters of brief presynaptic firing analysis

Table containing the number of analyzed pairs (n), mean, standard deviation (SD), median, p-values and used statistical tests of the short pulse analysis at all three different firing positions (pre, early, late). We did not observe any significant differences between both circuit motifs at all tested firing positions

	**Spike Loss (%)**					
**Time Slot**	Pre		Early		Late	
**n**	PV to SST 15	VIP to SST 20	PV to SST 15	VIP to SST 20	PV to SST 15	VIP to SST 20
**Mean**	13.42	8.172	22.86	12.20	21.91	12.85
**SD**	15.62	15.92	23.67	14.80	26.06	24.02
**Median**	0.000	0.000	16.67	6.667	20.00	6.000
**p**	0.7082		0.3620		0.6590	
**Statistical Test**	2way Anova (multiple comparison)		2way Anova (multiple comparison)		2way Anova (multiple comparison)	
	**ISI Increase (ms)**					
**Time Slot**	Pre		Early		Late	
**n**	PV to SST 15	VIP to SST 20	PV to SST 15	VIP to SST 20	PV to SST 15	VIP to SST 20
**Mean**	1.263	1.178	6.175	2.549	5.238	4.418
**SD**	1.825	2.818	8.428	3.413	6.365	9.597
**Median**	0.4230	0.3765	2.340	1.563	3.389	2.803
**p**	>0.9999		0.2386		0.9721	
**Statistical Test**	2way Anova (multiple comparison)		2way Anova (multiple comparison)		2way Anova (multiple comparison)	
	**Spike Loss/Number of Presynaptic Spikes (%)**					
**Time Slot**	Pre		Early		Late	
**n**	PV to SST 15	VIP to SST 20	PV to SST 15	VIP to SST 20	PV to SST 15	VIP to SST 20
**Mean**	0.5045	1.005	1.030	1.679	1.370	1.493
**SD**	0.6530	2.205	1.109	1.881	1.606	2.902
**Median**	0.000	0.000	0.6427	0.7044	0.9094	0.8319
**p**	0.7219		0.5098		0.9980	
**Statistical Test**	2way Anova (multiple comparison)		2way Anova (multiple comparison)		2way Anova (multiple comparison)	
