# Supplementary material for: Cortical PV and VIP interneurons similarly influence SST neuron output despite distinct unitary properties
Source: Commun Biol. 2026 Jun 3;9:756. doi: 10.1038/s42003-026-10418-2 (PMC13234287; doi:10.1038/s42003-026-10418-2)
Supplement: Supplementary file 3 — Description of Additional Supplementary Files [file 42003_2026_10418_MOESM3_ESM.pdf]

Description of Additional Supplementary Files

File name: Supplementary Data

Description Numerical source data underlying the graphs are provided as Numerical_Source_Data in the Supplemental data files
